# LOH, LST, and TAI Profiles in Solid Tumors Harboring Pathogenic or Likely Pathogenic *BRCA1/2* Variants: An Exploratory Study

**DOI:** 10.3390/ijms27198851

**Published:** 2026-10-04

**Authors:** Suleyman Erdogdu, Busra Ekinci, Seda Orenay-Boyacioglu, Olcay Boyacioglu, Ibrahim Halil Erdogdu, Ibrahim Meteoglu

**Affiliations:** 1 Department of Medical Oncology, Aydin City Hospital, Efeler, Aydin 09020, Türkiye; dr_suleyman21@hotmail.com; 2 Department of Medical Pathology, School of Medicine, Aydin Adnan Menderes University, Efeler, Aydin 09010, Türkiye; busra.ekinci@adu.edu.tr (B.E.); ibrahim.halil.erdogdu@adu.edu.tr (I.H.E.); imeteoglu@adu.edu.tr (I.M.); 3 Department of Medical Genetics, School of Medicine, Aydin Adnan Menderes University, Efeler, Aydin 09010, Türkiye; 4 Faculty of Engineering, Aydin Adnan Menderes University, Efeler, Aydin 09010, Türkiye; oboyaci@adu.edu.tr

**Keywords:** homologous recombination deficiency, *BRCA1*, *BRCA2*, genomic scar, LOH, LST, TAI

## Abstract

Homologous recombination deficiency (HRD) is a critical indicator of genomic instability; however, the composite HRD score may not reflect the distribution of distinct genomic scar components underlying this phenotype. In this exploratory study, we compared the absolute and relative contributions of loss of heterozygosity (LOH), large-scale state transitions (LST), and telomeric allelic imbalance (TAI) among solid tumors harboring pathogenic or likely pathogenic (P/LP) variants in *BRCA1* or *BRCA2*. This retrospective study included 76 patients with solid tumors who underwent HRD testing between January 2023 and January 2026. P/LP variants in homologous recombination repair (HRR) genes, alongside LOH, LST, and TAI scores, were evaluated. The composite HRD score was calculated as LOH + LST + TAI, with values ≥ 56 defined as HRD-positive. Additionally, the relative contribution of each scar component to the total HRD score was determined. Given the small sizes of the *BRCA1*-only and *BRCA2*-only subgroups and the heterogeneity of the overall cohort, all gene-group comparisons were considered exploratory and hypothesis-generating. Overall, 21.1% of the cohort was HRD-positive. Patients harboring *BRCA1/2* P/LP variants displayed a significantly higher median HRD score compared to non-carriers (47.0 vs. 30.0; *p* = 0.016). When comparing patients with tumors harboring *BRCA1*-only (*n* = 9) and *BRCA2*-only (*n* = 10) P/LP variants, LOH was higher in the *BRCA2* group (19.0 vs. 5.0; *p* = 0.036); however, this difference did not retain statistical significance following false discovery rate (FDR) adjustment (*q* = 0.108). In an exploratory sensitivity analysis restricted to breast and ovarian tumors (*BRCA1*-only, *n* = 5; *BRCA2*-only, *n* = 9), the LOH/HRD ratio was higher in the *BRCA2* group (0.301 vs. 0.000; *p* = 0.026; q = 0.039), whereas the LST/HRD ratio was higher in the *BRCA1* group (0.488 vs. 0.310; *p* = 0.019; q = 0.039). These estimates were based on very small subgroups and should therefore be interpreted cautiously. This exploratory analysis identified differences in the relative distributions of LOH and LST between tumors harboring *BRCA1*-only and *BRCA2*-only P/LP variants, particularly in the breast/ovarian subset. However, the small and heterogeneous subgroups, together with the absence of systematic assessment of biallelic *BRCA1/2* inactivation, preclude gene-specific or mechanistic conclusions. These observations should be regarded as hypothesis-generating and require validation in larger, tumor-specific, independent cohorts.

## 1. Introduction

Homologous recombination repair (HRR) plays a fundamental role in maintaining genomic integrity by ensuring high-fidelity repair of DNA double-strand breaks. Loss of function within this pathway leads to homologous recombination deficiency (HRD), driving increased genomic instability in tumor cells. HRD carries significant clinical importance, particularly due to its association with sensitivity to platinum-based chemotherapy and poly(ADP-ribose) polymerase (PARP) inhibitors [1,2,3]. Although initially identified predominantly in breast and ovarian cancers, the HRD phenotype is now recognized across diverse solid tumors, including prostate and pancreatic malignancies [1,2,3,4,5].

*BRCA1* and *BRCA2* are the genes most strongly associated with HRD; however, their respective roles in the homologous recombination process are not entirely identical. While *BRCA1* is involved in the DNA damage response, end resection, and pathway regulation, *BRCA2* functions specifically in executing *RAD51*-mediated homologous recombination. These functional distinctions are thought to leave distinct genomic imprints. Previous whole-genome studies incorporating evidence of gene inactivation have shown that *BRCA1*- and *BRCA2*-deficient tumors may exhibit different structural-variant patterns. In the CHORD study, *BRCA1* inactivation was associated with *BRCA1*-type HRD, whereas biallelic inactivation of *BRCA2*, *RAD51C*, and *PALB2* was predominantly associated with *BRCA2*-type HRD [6]. These findings suggest that HRD may not represent a uniform genomic phenotype.

Assessment of HRD relying solely on the detection of pathogenic alterations in HRR genes may be insufficient. The persistent alterations accumulated over time across the genome due to impaired DNA repair—termed genomic scars—provide complementary insights for defining the HRD phenotype [1,7]. The primary metrics utilized for this purpose include loss of heterozygosity (LOH), large-scale state transitions (LST), and telomeric allelic imbalance (TAI). Evaluating these three components in combination allows for the generation of a composite genomic instability/HRD score [7,8].

Although previous studies have demonstrated distinct genomic signatures in tumors with functionally established *BRCA1* or *BRCA2* deficiency [3,6], the relative contributions of LOH, LST, and TAI among tumors harboring BRCA1-only versus *BRCA2*-only P/LP variants remain insufficiently characterized. Furthermore, extensive pan-cancer studies have demonstrated that the genomic HRD phenotype does not completely overlap with *BRCA1/2* alteration status and that mechanisms beyond *BRCA1/2* can also generate prominent genomic scars [9,10,11,12,13]. These observations suggest that evaluating the individual components alongside the composite HRD score may provide additional descriptive information.

In this study, we evaluated *BRCA1/2* and other HRR gene alterations in conjunction with LOH, LST, and TAI profiles using HRD scores in a real-world cohort comprising diverse solid tumors. The aim of this exploratory study was to compare the absolute and relative contributions of LOH, LST, and TAI between tumors harboring *BRCA1*-only and *BRCA2*-only P/LP variants. In contrast to previous whole-genome and mutational-signature approaches that use genome-wide features and specialized computational classifiers to identify functional *BRCA1*- or *BRCA2*-type HRD patterns [3,6], the present study focused on the individual components already generated by a conventional composite HRD assay [2,7,8]. Thus, the incremental contribution of our analysis lies in examining whether LOH, LST, and TAI provide descriptive component-level information that may be obscured when HRD is reported only as a total score or binary classification. We did not assume that the presence of a P/LP variant was equivalent to biallelic *BRCA* inactivation or functional *BRCA*-driven HRD; rather, the analysis was intended to generate hypotheses for subsequent validation in molecularly characterized, tumor-specific cohorts.

## 2. Results

### 2.1. Patient Characteristics and HRD Distribution

A total of 76 patients with solid tumors who underwent HRD testing between January 2023 and January 2026 were included in the study. The cohort comprised 62 females (81.6%) and 14 males (18.4%). The mean age was 57.2 ± 11.7 years, with a median age of 58 years (interquartile range (IQR): 51–65). Ovarian cancer accounted for half of the cohort (38/76, 50.0%), followed by breast (15/76, 19.7%) and prostate cancers (10/76, 13.2%).

Across the entire cohort, the mean composite HRD score was 35.6 ± 24.7, and the median HRD score was 33.0 (IQR: 14.5–51.3). Based on the HRD cutoff threshold of ≥56, 16 patients (21.1%) were classified as HRD-positive and 60 patients (78.9%) as HRD-negative. When evaluating individual genomic scar components, the median LOH, LST, and TAI values were 4.5, 11.5, and 14.0, respectively. Demographic characteristics, tumor-type distribution, and genomic scar parameters of the study cohort are summarized in Table 1.

### 2.2. Tumors Harboring BRCA1/2 P/LP Variants and Genomic Instability

*BRCA1* and/or *BRCA2* P/LP variants were identified in 21 patients. Among these, nine patients harbored *BRCA1*-only alterations, 10 had *BRCA2*-only alterations, and two patients carried P/LP variants in both genes. Patient-level characteristics of the 21 tumors harboring *BRCA1/2* P/LP variants, including tumor type, affected gene, variant classification, HRD status, composite HRD score, and individual LOH, LST, and TAI values, are presented in Supplementary Table S1.

**Figure 1:**
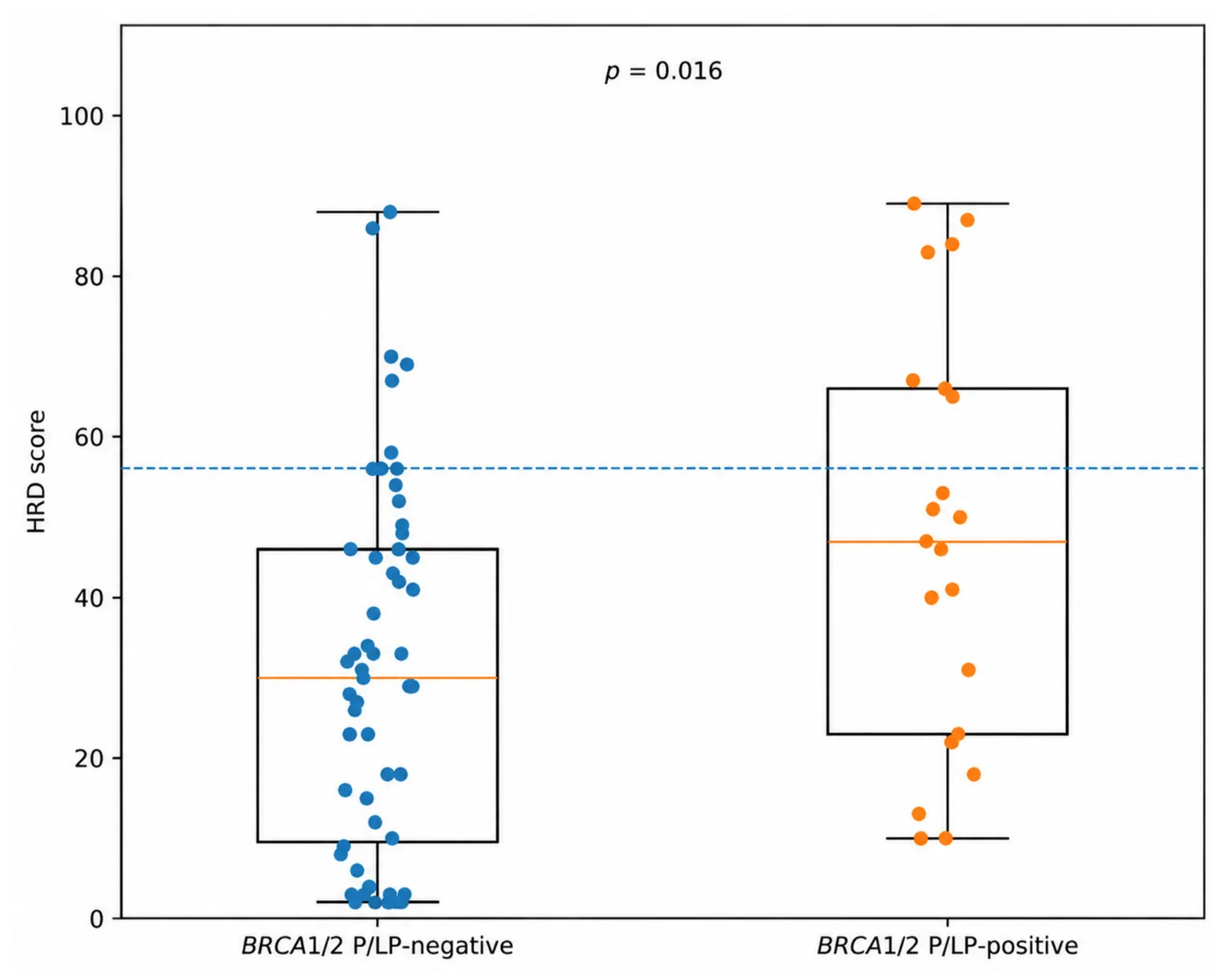
HRD score according to *BRCA1/2* P/LP variant status. The dashed horizontal line indicates the predefined HRD positivity threshold (HRD score ≥ 56). Individual points represent patients; boxes indicate the distribution of HRD scores.

Patients harboring *BRCA1/2* P/LP variants exhibited significantly higher overall genomic instability compared to *BRCA1/2* P/LP-negative patients. The median HRD score in the BRCA1/2 P/LP variant-positive group was 47.0 (IQR: 23.0–66.0), whereas it was 30.0 (IQR: 9.5–46.0) in the *BRCA1/2* P/LP-negative group (*p* = 0.016). A comparison of composite HRD scores and individual genomic scar components between carriers and non-carriers of *BRCA1/2* P/LP variants is presented in Table 2. The distribution of HRD scores stratified by *BRCA1/2* status is also illustrated in Figure 1.

Patients harboring *BRCA1/2* P/LP variants exhibited nominally higher LST and TAI values than *BRCA1/2* P/LP variant-negative patients. However, neither difference remained statistically significant after FDR adjustment (q = 0.063 for both comparisons). The median LST score was 14.0 in the *BRCA1/2* group compared to 10.0 in the *BRCA1/2* P/LP-negative group (*p* = 0.029). Similarly, median TAI scores were 19.0 and 11.0, respectively (*p* = 0.042). Although a trend in the same direction was observed for LOH, the difference between the median of 7.0 in the *BRCA1/2* group and 3.0 in the P/LP-negative group did not reach statistical significance (*p* = 0.064).

A higher rate of HRD positivity was also observed in the *BRCA1/2* P/LP variant-positive group. Seven out of 21 patients (33.3%) with *BRCA1/2* P/LP variants were HRD-positive, compared to 9 out of 55 patients (16.4%) in the *BRCA1/2* P/LP-negative group. Although the odds ratio (OR) for HRD positivity in the presence of tumors harboring *BRCA1/2* P/LP variants was 2.56, this difference did not achieve statistical significance (OR = 2.56; *p* = 0.124).

### 2.3. Comparison of Genomic Scar Profiles Between the BRCA1-Only and BRCA2-Only P/LP Variant Groups

To explore potential differences in genomic scar-component distributions, nine patients harboring *BRCA1*-only P/LP variants were compared with ten patients harboring *BRCA2*-only P/LP variants. Although the median composite HRD score was higher in the *BRCA2* group, the difference between groups was not statistically significant (median 56.0 vs. 41.0; *p* = 0.253).

The most pronounced difference between the *BRCA1* and *BRCA2* groups was observed in LOH levels. The median LOH value was 19.0 in the *BRCA2* group compared to 5.0 in the *BRCA1* group (*p* = 0.036). However, following Benjamini–Hochberg correction for multiple testing, this association did not retain statistical significance (FDR q = 0.108). While the relative contribution of LOH to the composite HRD score trended higher in the *BRCA2* group (0.297 vs. 0.140; *p* = 0.065; q = 0.099), the relative contribution of LST was higher in the *BRCA1* group (0.419 vs. 0.329; *p* = 0.066; q = 0.099). Neither difference reached statistical significance following FDR adjustment.

**Figure 2:**
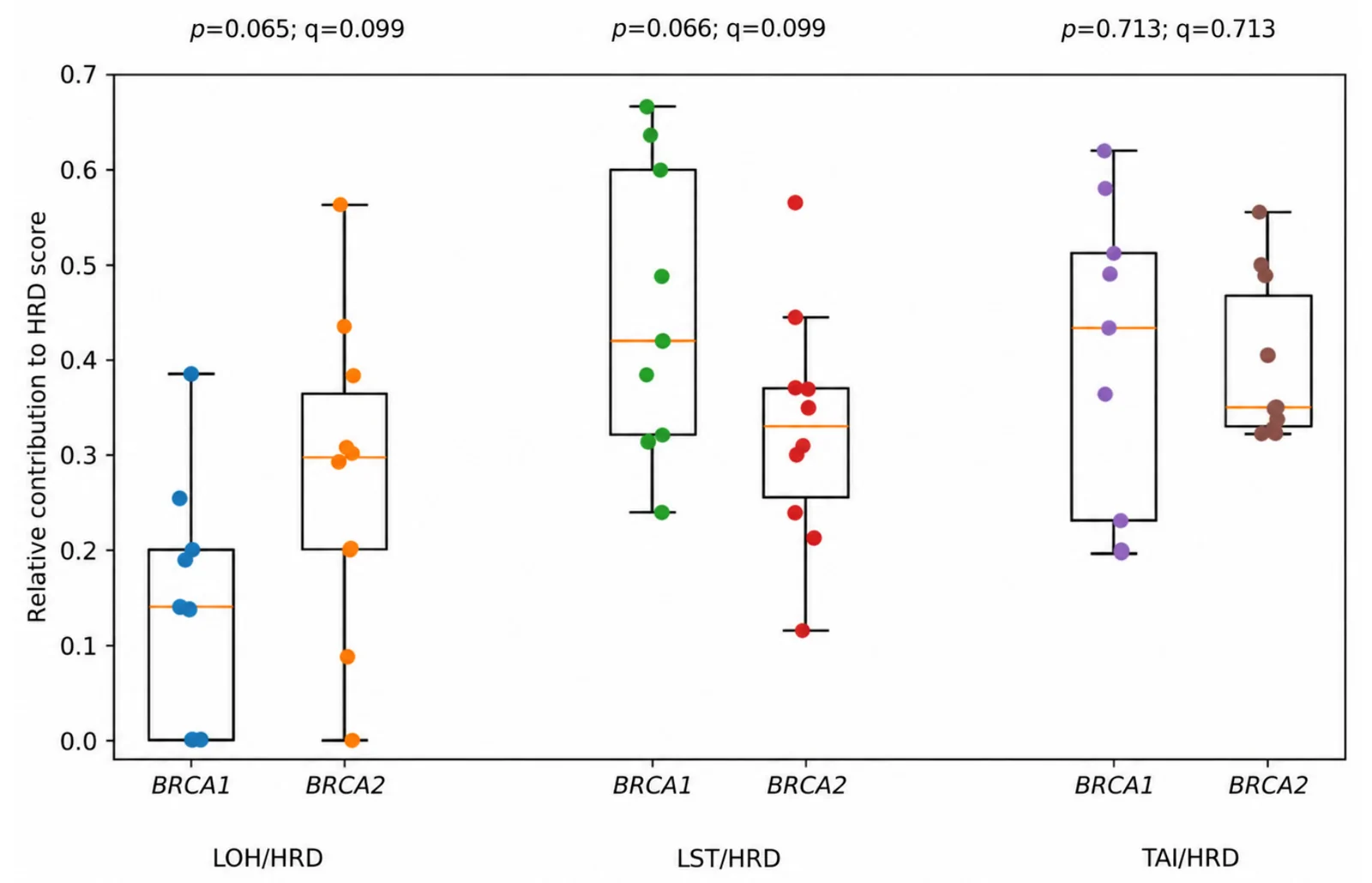
Relative genomic scar composition in tumors harboring *BRCA1*-only and *BRCA2*-only P/LP variants.

Absolute and relative genomic scar profiles of *BRCA1*-only and *BRCA2*-only P/LP variant groups are compared in Table 3. Differences in the relative contributions of genomic scars to the composite HRD score are shown in Figure 2.

### 2.4. Exploratory Breast/Ovarian Sensitivity Analysis of the BRCA1-Only and BRCA2-Only Groups

Because tumor-type heterogeneity could confound comparisons between the *BRCA1*-only and *BRCA2*-only groups, an exploratory sensitivity analysis was restricted to breast and ovarian tumors, which constituted the most represented clinically relevant tumor subset. This analysis included five patients with *BRCA1*-only P/LP variants and nine with *BRCA2*-only P/LP variants. Owing to these very small subgroup sizes, the analysis was considered hypothesis-generating and was not intended to establish stable gene-specific genomic scar patterns.

In this analysis, the distribution of genomic scars within the composite HRD score revealed a more distinct divergence compared to the pan-tumor analysis. In the sensitivity analysis restricted to breast and ovarian tumors, the LOH/HRD ratio was significantly higher in the *BRCA2* group compared to the *BRCA1* group (0.301 vs. 0.000; *p* = 0.026), whereas the LST/HRD ratio was significantly higher in the *BRCA1* group (0.488 vs. 0.310; *p* = 0.019). No significant difference was observed for the TAI/HRD ratio (*p* = 0.518; q = 0.518). Both associations retained significance after Benjamini–Hochberg correction across the three genomic scar composition parameters (q = 0.039 for LOH/HRD; q = 0.039 for LST/HRD).

The divergence in genomic scar composition observed in the sensitivity analysis of breast and ovarian tumors was accompanied by large effect sizes. The LOH/HRD ratio was higher in the *BRCA2*-only group compared to the *BRCA1*-only group (0.301 vs. 0.000; *p* = 0.026; q = 0.039), yielding a Cliff’s δ = 0.756 (95% CI: 0.111–1.000). Conversely, the LST/HRD ratio was higher in the *BRCA1*-only group (0.488 vs. 0.310; *p* = 0.019; q = 0.039), with a Cliff’s δ calculated in the *BRCA2*–*BRCA1* direction of −0.778 (95% CI: −1.000 to −0.022). No significant difference was detected for TAI/HRD (*p* = 0.518; q = 0.518; δ = −0.244, 95% CI: −0.822 to 0.556). Although the point estimates for Cliff’s delta were large, the corresponding confidence intervals were wide, reflecting substantial uncertainty arising from the very small subgroup sizes. Accordingly, the effect-size estimates should be interpreted as descriptive and exploratory rather than as evidence of stable *BRCA1*- or *BRCA2*-specific effects.

**Figure 3:**
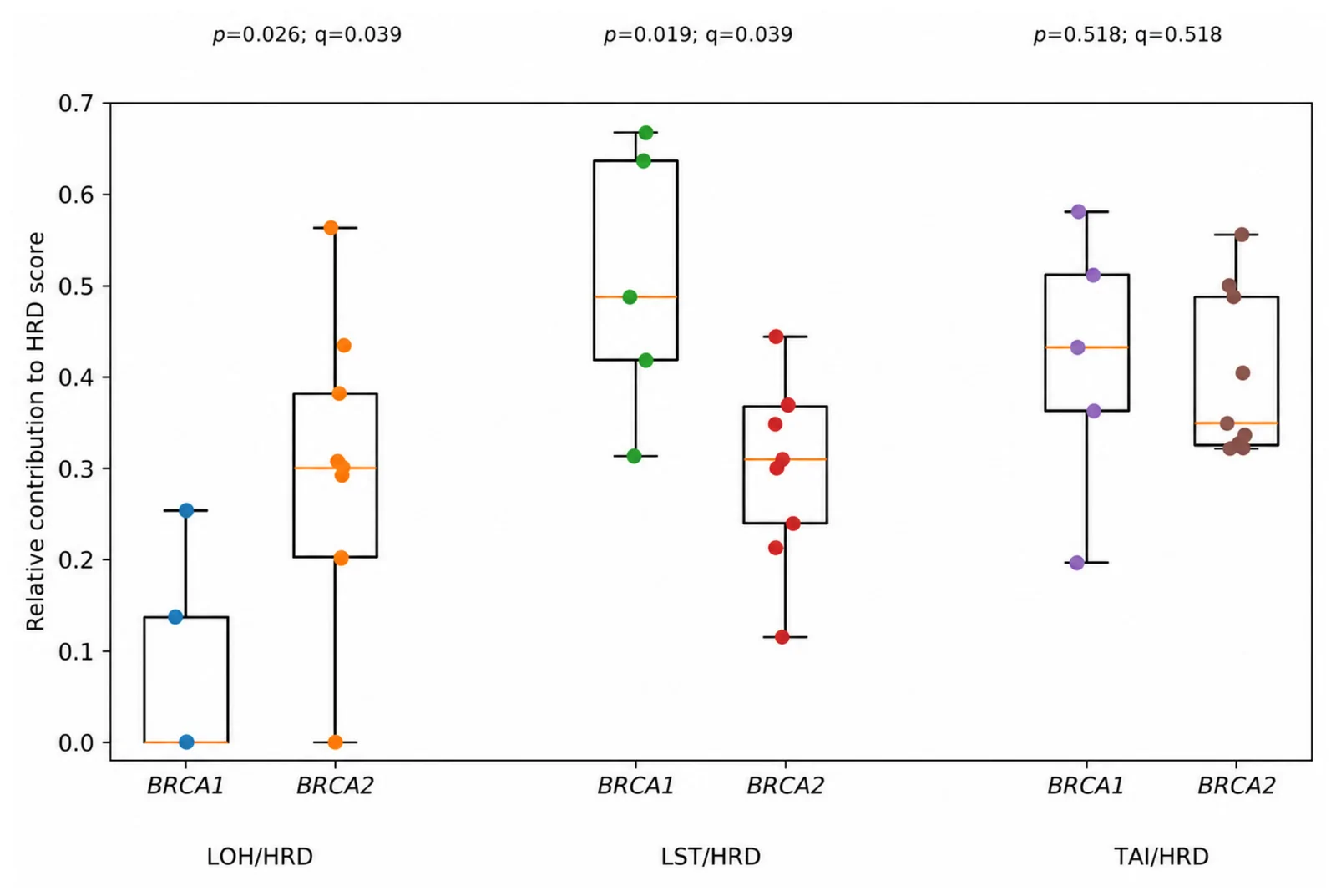
Genomic Scar Composition in Breast and Ovarian Tumors.

The observed differences in genomic scar-component distributions in the exploratory analysis restricted to breast and ovarian tumors are illustrated in Figure 3, with the corresponding comparisons presented in Table 4.

### 2.5. Post Hoc Robustness Analysis Restricted to Ovarian Tumors

The breast/ovarian subset was not compositionally balanced: all five BRCA1-only tumors were ovarian, whereas the BRCA2-only group comprised six ovarian and three breast tumors. Therefore, residual confounding by tumor type could not be excluded. To further examine this issue, a post hoc robustness analysis was restricted to ovarian tumors, including five tumors harboring BRCA1-only P/LP variants and six harboring BRCA2-only P/LP variants.

The direction of the component differences was preserved. The median LOH/HRD ratio was higher in the BRCA2-only group than in the BRCA1-only group (0.338 vs. 0.000; *p* = 0.082; q = 0.123), whereas the median LST/HRD ratio was higher in the BRCA1-only group (0.488 vs. 0.270; *p* = 0.030; q = 0.091). The median TAI/HRD ratio did not differ between the groups (0.433 vs. 0.371; *p* = 0.662; q = 0.662). However, none of these comparisons remained statistically significant after FDR adjustment. Thus, the directions were consistent with those observed in the combined breast/ovarian analysis, but statistical precision was reduced because of the very small subgroup sizes. A breast-only comparison was not performed because no BRCA1-only P/LP variant-positive breast tumors were available (Supplementary Table S2).

### 2.6. HRR Genes and HRD Positivity

When individual HRR genes included in the panel were examined, the strongest association with HRD positivity was observed for *BARD1*. Five out of seven patients (71.4%) with a *BARD1* P/LP variant were HRD-positive, compared to 11 out of 69 patients (15.9%) without *BARD1* alterations. *BARD1* alterations were associated with significantly increased odds of HRD positivity (OR = 13.18; *p* = 0.0038). However, following Benjamini–Hochberg correction across the 15 panel genes, this association did not retain statistical significance (FDR q = 0.056).

A similar but weaker association was observed for *BRCA2*. Six of 12 patients (50.0%) with *BRCA2* P/LP variants were HRD-positive, compared to 10 of 64 patients (15.6%) without *BRCA2* alterations (OR = 5.40; *p* = 0.015). However, this relationship did not maintain significance after FDR adjustment across the 15 genes (q = 0.114). Exploratory associations between individual HRR genes and HRD positivity are depicted in Supplementary Table S3 and Supplementary Figure S1.

### 2.7. Concordance Between Tumors Harboring BRCA1/2 P/LP Variants and Genomic HRD Phenotype

Concordance between *BRCA1/2* genotype and the genomic HRD phenotype was incomplete across individual patients. Among the 16 HRD-positive patients, seven (43.8%) harbored a *BRCA1* and/or *BRCA2* P/LP variant, whereas nine (56.3%) were negative for *BRCA1/2* P/LP variants. Conversely, 14 out of 21 patients (66.7%) carrying *BRCA1/2* P/LP variants displayed an HRD score below 56. These findings demonstrate a discrepancy between *BRCA1/2* genotype status and genomic scar-based HRD classification within this cohort.

**Figure 4:**
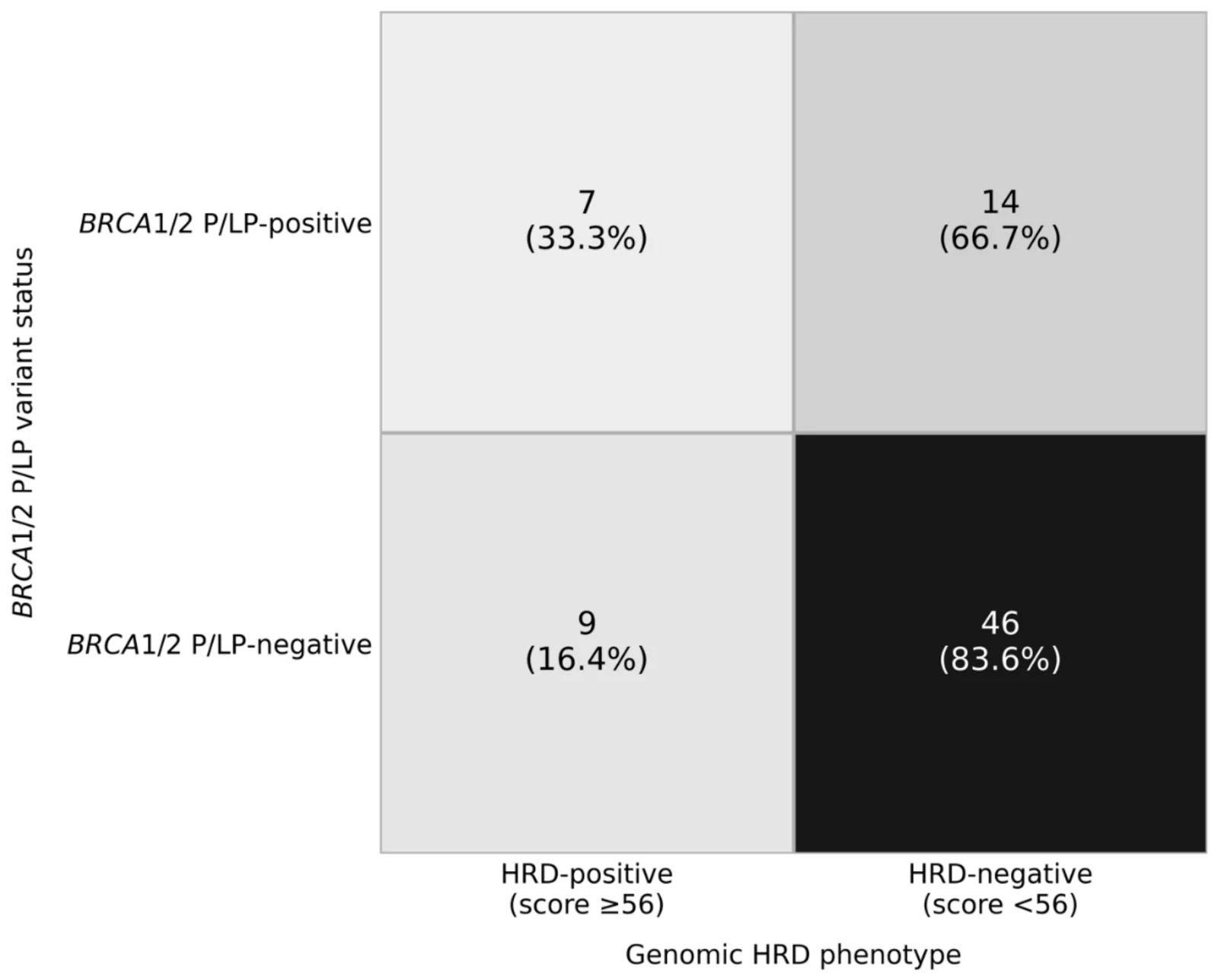
Concordance Between *BRCA1/2* P/LP Variant Status and Genomic HRD Phenotype.

Further evaluation of the nine *BRCA1/2* P/LP-negative, HRD-positive patients revealed *ATM* and *CHEK2* alterations in five patients each, and *PALB2* and *CHEK1* alterations in three patients each. Within the *BRCA1/2* P/LP-negative subgroup, 5 of 12 patients carrying an *ATM* P/LP variant were HRD-positive, compared to only 4 of 43 patients without *ATM* alterations (OR ≈ 6.96; *p* ≈ 0.017). However, upon applying multiple testing corrections for non-*BRCA* HRR genes, this association lost statistical significance and was considered exploratory. The relationship between BRCA1/2 P/LP variant status and the genomic HRD phenotype is summarized in Supplementary Table S2 and illustrated in Figure 4.

## 3. Discussion

In this real-world cohort, tumors harboring *BRCA1/2* P/LP variants had higher composite HRD scores than tumors without such variants. The exploratory comparison of the *BRCA1*-only and *BRCA2*-only groups identified nominal differences in selected genomic scar components; however, the pan-tumor LOH difference did not remain significant after FDR correction. In the combined breast/ovarian sensitivity analysis, the LOH/HRD ratio was higher in the *BRCA2*-only group, whereas the LST/HRD ratio was higher in the *BRCA1*-only group, with both differences remaining statistically significant after FDR adjustment. However, this subgroup was compositionally unbalanced: all five BRCA1-only tumors were ovarian, whereas the BRCA2-only group included six ovarian and three breast tumors. Therefore, residual confounding by tumor type could not be excluded. In a post hoc robustness analysis restricted to ovarian tumors, the directions of the LOH/HRD and LST/HRD differences were preserved, but neither remained statistically significant after FDR adjustment. This loss of statistical significance should be interpreted in the context of the very small ovarian-only subgroup sizes rather than as evidence of an effect in the opposite direction. Collectively, these findings provide directional consistency but should be considered preliminary and hypothesis-generating rather than evidence of stable gene-specific genomic scar patterns.

These findings should be interpreted in relation to, rather than as a replacement for, previous whole-genome and mutational-signature studies. The CHORD classifier developed by Nguyen et al. used genome-wide mutational and structural-variant features to distinguish *BRCA1*-type from *BRCA2*-type HRD and incorporated evidence of gene inactivation [6]. Similarly, in a pan-cancer analysis of 8847 tumors by Rempel et al., the HRDsum score comprising LOH, LST, and TAI was associated with *BRCA*/HRR genotype, particularly in tumors with biallelic *BRCA1/2* loss [14]. Unlike these approaches, the present study does not seek to infer functional *BRCA1*- or *BRCA2*-type HRD. Its more limited and complementary contribution lies in examining whether the individual components already generated by a conventional HRD assay provide descriptive information beyond the composite score and binary HRD classification. Accordingly, the observed differences should be regarded as preliminary component-level patterns rather than evidence of distinct functional *BRCA1*- and *BRCA2*-deficiency states.

A key advantage of the composite HRD score is its ability to measure the cumulative footprint left across the genome by HRR deficiency over time; however, this same property can also present a limitation. LOH, LST, and TAI represent distinct chromosomal events, and aggregating them into a single metric may obscure the relative weight of each individual component. Stewart et al. similarly emphasized that genetic etiology and genomic consequences are not identical concepts in HRD assessment, noting that different testing platforms measure distinct aspects of HRD [2]. Contemporary evaluations of HRD assays also highlight that while genomic scars are clinically useful, marked variations exist among assays regarding algorithms, thresholds, and biological scope [7,15]. In this regard, our study offers an exploratory framework suggesting that examining the internal architecture of genomic scars without altering the composite HRD score may yield incremental biological insight.

Furthermore, our study revealed an incomplete overlap between the genomic HRD phenotype and *BRCA1/2* genotype. Notably, 9 out of 16 HRD-positive patients (56.3%) lacked *BRCA1/2* P/LP variants. Conversely, 14 out of 21 patients carrying *BRCA1/2* P/LP variants exhibited HRD scores below 56. This discordance is well documented in the literature. Recently, evaluating 580 tumor–normal matched samples via whole-genome sequencing in 2026, Al Assaad et al. demonstrated that 24% of HRD-positive tumors were *BRCA1/2* wild-type [16]. Similarly, in the study by Rempel et al., HRD positivity was observed across a much broader spectrum of tumors than those harboring deleterious *BRCA1/2* variants [14]. Taken together, these data indicate that *BRCA1/2* P/LP variant status may be associated with genomic HRD but is neither necessary nor sufficient to define the genomic HRD phenotype.

Our findings regarding non-*BRCA* HRR genes further reinforce this heterogeneity. Although *BARD1* alterations demonstrated the strongest nominal association with HRD positivity, statistical significance was not retained following FDR correction across the 15 panel genes. Consequently, it is not possible to conclude that *BARD1* is an independent determinant of HRD. Indeed, a study of 483 patients reported that only 27.3% of cases harboring non-*BRCA1/2* HRR variants were HRD-positive, with strong associations reported for genes such as *PALB2*, *RAD51C/D*, and *RAD54L*, but weaker associations for *ATM*, *BRIP1*, and *CDK12* [17]. This highlights that not all HRR genes induce a genomic HRD phenotype to the same extent, and gene-level results must be interpreted alongside tumor type, allelic status, and the functional impact of individual variants.

Several aspects of this study enhance the relevance of our findings. First, HRD was not evaluated solely as a binary positive/negative classification or through the composite HRD score alone; LOH, LST, and TAI components were dissected individually, and the relative contribution of each component to the total score was calculated at the individual patient level. This approach allowed for direct investigation of the hypothesis that comparable composite HRD scores may arise from distinct genomic scar compositions. Second, rather than pooling *BRCA1* and *BRCA2* alterations into a single *BRCA*-positive category, cases harboring *BRCA1*-only and *BRCA2*-only P/LP variants were evaluated separately. In this respect, our study presents an alternative approach to exploring genome-wide findings—which established that *BRCA1*-type and *BRCA2*-type HRD exhibit distinct genomic characteristics [6]—through more routinely accessible LOH, LST, and TAI measurements. Furthermore, to account for potential confounding by tumor-type heterogeneity inherent to a pan-tumor cohort, a sensitivity analysis restricted to breast and ovarian malignancies was conducted. The combined breast/ovarian sensitivity analysis reduced the overall heterogeneity of the pan-tumor cohort; however, the absence of breast tumors in the *BRCA1*-only group meant that residual tumor-type confounding could not be eliminated. The preservation of the effect directions in the post hoc ovarian-only analysis provides directional consistency, although the absence of FDR-adjusted significance precludes a confirmatory interpretation. Finally, applying the Benjamini–Hochberg FDR correction alongside nominal *p*-values, and treating *BARD1* and pan-tumor LOH findings as exploratory after losing significance post-correction, mitigates the risk of overinterpreting false-positive results in a study with modest sample size.

Despite these strengths, our findings should be interpreted within the context of several limitations. First, the retrospective, single-center design combined with a limited total sample size reduces statistical power, particularly for comparisons between the *BRCA1*-only and *BRCA2*-only subgroups. Because sample sizes in the breast and ovarian sensitivity analysis were even smaller, results retaining significance post-FDR should be regarded as exploratory rather than confirmatory. Additionally, due to the pan-tumor nature of the cohort, tumor-type-specific biological variations cannot be completely ruled out. It should also be noted that key variables capable of influencing the HRD phenotype—such as germline vs. somatic origin, biallelic inactivation, locus-specific LOH, promoter methylation, and prior treatment history—could not be systematically evaluated across all cases. Given that recent pan-cancer studies demonstrate a substantially stronger association between biallelic gene loss and HRD [4,14], incorporating this variable into future analyses will be essential. The effective sample size for the central comparison was substantially smaller than the total cohort size, comprising only nine *BRCA1*-only and ten *BRCA2*-only tumors. The breast/ovarian sensitivity analysis was further restricted to five and nine cases, respectively. These small and unbalanced subgroups limit statistical power, produce imprecise effect-size estimates, and increase the possibility that the observed differences reflect sampling variability. Multiple-testing correction reduces but does not eliminate this concern. Therefore, the analyses cannot establish robust or generalizable gene-specific genomic scar patterns.

## 4. Materials and Methods

### 4.1. Study Design and Patient Selection

This study was approved by the Institutional Non-Interventional Clinical Research Ethics Committee (Approval No: 2026/#253, Date: 2 July 2026). The study was conducted retrospectively, and all procedures adhered to the principles of the Declaration of Helsinki. Patient data were anonymized prior to analysis.

Patients diagnosed with solid tumors who underwent HRD analysis as part of molecular evaluation between January 2023 and January 2026 were included in this retrospective study. Patient age, sex, primary tumor site, and histopathological diagnosis were retrospectively retrieved from medical records. Completion of HRD testing and the availability of complete data for composite HRD score along with LOH, LST, and TAI values were set as the primary inclusion criteria. Samples with insufficient tumor tissue for molecular analysis or those failing quality control criteria were excluded. Based on these criteria, 76 patients were included in the final analysis.

### 4.2. Evaluation of Tumor Tissue and DNA Isolation

Molecular analyses were performed using formalin-fixed, paraffin-embedded (FFPE) tumor tissue. Prior to analysis, hematoxylin and eosin-stained sections were evaluated by a pathologist, and regions with adequate tumor cellularity were selected for molecular testing.

Genomic DNA was isolated using the QIAamp DNA FFPE Tissue Kit (QIAGEN, Hilden, Germany) in accordance with the protocol described by Cokpinar et al. (2024) [18]. Following isolation, DNA concentration and purity were assessed spectrophotometrically. Samples displaying an OD_260_/OD_280_ ratio between 1.8 and 2.0 were considered suitable for downstream molecular analysis.

### 4.3. HRD Panel and Next-Generation Sequencing

The HRD Panel Score CDHS-50893Z (QIAGEN, Hilden, Germany) was employed to evaluate genetic alterations in the HRR pathway and HRD-associated genomic instability. The panel targeted 15 genes within the HRR pathway using a total of 16,173 primers. The analyzed genes included *ATM*, *BARD1*, *BRCA1*, *BRCA2*, *BRIP1*, *CDK12*, *CHEK1*, *CHEK2*, *FANCL*, *PALB2*, *PPP2R2A*, *RAD51B*, *RAD51C*, *RAD51D*, and *RAD54L*.

Following genomic DNA isolation and quality control, target regions were prepared and sequencing libraries were constructed according to the manufacturer’s protocol. Next-generation sequencing was subsequently performed on the MiSeq System (Illumina, San Diego, CA, USA). The mean sequencing depth across target regions was approximately 3000×. A minimum variant allele frequency threshold of 1% was applied for variant calling in tumor samples. Sequencing data failing to meet designated quality control metrics and alterations lacking support for reliable variant calling were excluded from downstream analyses.

### 4.4. Determination of Genomic Scars and HRD Score

In addition to HRR gene variants, the three primary genomic scar components resulting from HRD—LOH, LST, and TAI—were evaluated individually for each tumor sample. LOH represents the loss of one allele in heterozygous genomic regions; LST denotes structural transitions between large chromosomal segments; and TAI refers to regions of allelic imbalance extending to the telomere without crossing the centromere [7,8]. For each patient, the composite HRD score was calculated as the sum of LOH, LST, and TAI values. According to the panel’s evaluation algorithm, samples with an HRD score of 56 or higher (≥56) were classified as HRD-positive, whereas those with scores below 56 (<56) were designated as HRD-negative. In addition to categorical classification, the HRD score was analyzed as a continuous variable to preserve patient-level granularity in genomic instability levels.

In line with the primary objective of this study, not only the magnitude of the composite HRD score but also the relative distribution of its constituent genomic scars was investigated. To this end, the relative contribution of each scar component was calculated for each patient by dividing the respective score by the total HRD score (i.e., LOH/HRD, LST/HRD, and TAI/HRD). This enabled us to evaluate whether comparable overall HRD levels in *BRCA1*-only and *BRCA2*-only P/LP variant groups stemmed from distinct LOH, LST, and TAI compositions.

### 4.5. Bioinformatic Analysis and Variant Classification

Data analysis and variant interpretation following sequencing were conducted using QIAGEN Clinical Insight Interpret (QCI™; QIAGEN, Hilden, Germany) software (v8.1.202021). Variants passing quality control filters were classified according to their clinical significance based on the American College of Medical Genetics and Genomics (ACMG) guidelines.

Only variants classified as pathogenic (P) or likely pathogenic (LP) were included in comparative genomic and statistical analyses. Although variants of uncertain significance were documented, they were excluded from downstream comparative analyses due to their unconfirmed biological and clinical impact. Benign and likely benign variants were also omitted from evaluation.

Patients were stratified into molecular subgroups based on the presence of P/LP variants in *BRCA1*, *BRCA2*, and non-*BRCA* HRR genes. For the direct comparison of genomic scar profiles between the *BRCA1*-only and *BRCA2*-only P/LP variant groups, the two patients harboring concurrent P/LP variants in both genes were excluded only from this specific comparison but retained in analyses of the overall *BRCA1/2* P/LP variant-positive group.

### 4.6. Statistical Analysis

Distribution characteristics of continuous variables were evaluated using normality tests and graphical methods. Normally distributed data were presented as mean ± standard deviation, whereas non-normally distributed data were expressed as median and IQR. Categorical variables were reported as counts and percentages. HRD status was evaluated categorically as HRD-positive (≥56) and HRD-negative (<56) based on the predefined cutoff of 56, while the composite HRD score was also analyzed as a continuous variable.

Differences in composite HRD score as well as LOH, LST, and TAI values between *BRCA1* and *BRCA2* groups were evaluated using the Mann–Whitney U test, taking into account sample sizes and distribution characteristics. The Kruskal–Wallis test was applied when comparing more than two independent molecular groups. HRD positivity rates and other categorical variables were compared using the Chi-square test or Fisher’s exact test, depending on expected cell counts.

To assess the magnitude of group differences in small *BRCA1* and *BRCA2* subgroups, Cliff’s delta (δ) effect size was calculated in addition to Mann–Whitney U tests. Corresponding 95% confidence intervals for effect sizes were generated using a bias-corrected and accelerated (BCa) bootstrap method based on 9999 resamplings.

Multiple testing corrections were applied across distinct analysis families corresponding to biologically related hypothesis groups. Absolute genomic scar components (LOH, LST, and TAI) and relative scar ratios (LOH/HRD, LST/HRD, and TAI/HRD) were evaluated as separate test families. For gene-level analyses, the 15 HRR genes on the panel were defined as a single test family. Benjamini–Hochberg adjusted q-values < 0.05 were considered statistically significant.

To further examine residual confounding by tumor type, a post hoc robustness analysis was restricted to ovarian tumors because the breast subset contained no *BRCA1*-only P/LP variant-positive tumors. Relative LOH/HRD, LST/HRD, and TAI/HRD ratios were compared using exact two-sided Mann–Whitney U tests, with Benjamini–Hochberg adjustment across the three comparisons. A breast-only between-group analysis was not performed because no *BRCA1*-only P/LP variant-positive breast tumors were available.

## 5. Conclusions

In this exploratory cohort, tumors harboring *BRCA1*-only and *BRCA2*-only P/LP variants showed preliminary differences in the relative distributions of selected genomic scar components. The combined breast/ovarian sensitivity analysis suggested a greater LOH contribution in the *BRCA2*-only group and a greater LST contribution in the *BRCA1*-only group. Although the directions of these differences were preserved in the post hoc ovarian-only analysis, neither remained significant after FDR adjustment. Accordingly, the small subgroup sizes, residual tumor-type heterogeneity, and lack of systematic assessment of biallelic *BRCA1/2* inactivation preclude gene-specific or mechanistic conclusions. These observations require validation in larger, independent, tumor-specific cohorts with tumor–normal sequencing, locus-specific LOH assessment, and systematic characterization of biallelic *BRCA1/2* loss.

## Figures and Tables

**Table 1:** Clinicopathological and HRD characteristics of the study cohort.

Characteristic		Overall Cohort (*n* = 76)
Age, years	Mean ± SD	57.2 ± 11.7
	Median (IQR)	58 (51–65)
Sex, *n* (%)	Female	62 (81.6)
	Male	14 (18.4)
Primary tumor site, *n* (%)	Ovary	38 (50.0)
	Breast	15 (19.7)
	Prostate	10 (13.2)
	Pancreas	3 (3.9)
	Endometrium	2 (2.6)
	Colon	2 (2.6)
	Cervix uteri	1 (1.3)
	Rectum	1 (1.3)
	Lung	1 (1.3)
	Small intestine	1 (1.3)
	Corpus uteri	1 (1.3)
	Biliary tract	1 (1.3)
HRD status, *n* (%)	HRD-positive (score ≥ 56)	16 (21.1)
	HRD-negative (score < 56)	60 (78.9)
HRD score	Mean ± SD	35.6 ± 24.7
	Median (IQR)	33.0 (14.5–51.3)
Genomic scar components, median (IQR)	LOH	4.5 (1.0–14.0)
	LST	11.5 (6.0–19.0)
	TAI	14.0 (5.0–22.0)

**Table 2:** Genomic scar burden according to *BRCA1/2* P/LP variant status.

Variable	*BRCA1/2* P/LP (*n* = 21)	*BRCA1/2* P/LP-Negative (*n* = 55)	*p*-Value	FDR q
HRD score	47.0 (23.0–66.0)	30.0 (9.5–46.0)	0.016	—
LOH	7.0 (2.0–19.0)	3.0 (1.0–8.0)	0.064	0.064
LST	14.0 (10.0–24.0)	10.0 (4.5–18.0)	0.029	0.063
TAI	19.0 (10.0–28.0)	11.0 (3.0–21.0)	0.042	0.063
HRD-positive (≥56), *n* (%)	7 (33.3)	9 (16.4)	0.124	—

*p* values were adjusted for multiple comparisons using the Benjamini–Hochberg FDR method.

**Table 3:** Comparison of genomic scar profiles between tumors harboring *BRCA1*-only and *BRCA2*-only P/LP variants.

Variable	*BRCA1*-Only (*n* = 9)	*BRCA2*-Only (*n* = 10)	*p*-Value	FDR q
HRD score	41.0 (22.0–51.0)	56.0 (28.8–83.8)	0.253	—
LOH	5.0 (0.0–7.0)	19.0 (5.8–23.8)	0.036	0.108
LST	14.0 (12.0–20.0)	12.0 (10.0–25.5)	0.935	0.935
TAI	18.0 (8.0–26.0)	20.0 (11.2–28.8)	0.487	0.730
LOH/HRD	0.140 (0.000–0.200)	0.297 (0.201–0.364)	0.065	0.099
LST/HRD	0.419 (0.321–0.600)	0.329 (0.254–0.370)	0.066	0.099
TAI/HRD	0.433 (0.231–0.512)	0.349 (0.329–0.467)	0.713	0.713

*p* values were adjusted for multiple comparisons using the Benjamini–Hochberg FDR method.

**Table 4:** Breast/ovarian sensitivity analysis of genomic scar composition.

Variable	*BRCA1* Only (*n* = 5)	*BRCA2* Only (*n* = 9)	*p*-Value	FDR q
LOH/HRD	0.000 (0.000–0.137)	0.301 (0.202–0.383)	0.026	0.039
LST/HRD	0.488 (0.419–0.636)	0.310 (0.239–0.369)	0.019	0.039
TAI/HRD	0.433 (0.364–0.512)	0.349 (0.326–0.488)	0.518	0.518

*p* values were adjusted for multiple comparisons using the Benjamini–Hochberg FDR method.

## Data Availability

The datasets generated and analyzed during the current study are not publicly available due to patient privacy and ethical restrictions regarding sensitive genomic and clinical data, but are available from the corresponding author on reasonable request.

## Supplementary Materials

The following supporting information can be downloaded at: https://www.mdpi.com/article/10.3390/ijms27198851/s1.

## Abbreviations

The following abbreviations are used in this manuscript:

CI	Confidence interval
FDR	False discovery rate
FFPE	Formalin-fixed paraffin-embedded
HRD	Homologous recombination deficiency
HRR	Homologous recombination repair
IQR	Interquartile range
LOH	Loss of heterozygosity
LP	Likely pathogenic
LST	Large-scale state transitions
OR	Odds ratio
P	Pathogenic
SD	Standard deviation
TAI	Telomeric allelic imbalance
