# Supplementary material for: LOH, LST, and TAI Profiles in Solid Tumors Harboring Pathogenic or Likely Pathogenic BRCA1/2 Variants: An Exploratory Study
Source: Int J Mol Sci. 2026 Oct 4;27(19):8851. doi: 10.3390/ijms27198851 (PMC13664783; doi:10.3390/ijms27198851)
Supplement: Supplementary file 1 [file ijms-27-08851-s001.zip › Supplementary Figure S1.pdf]

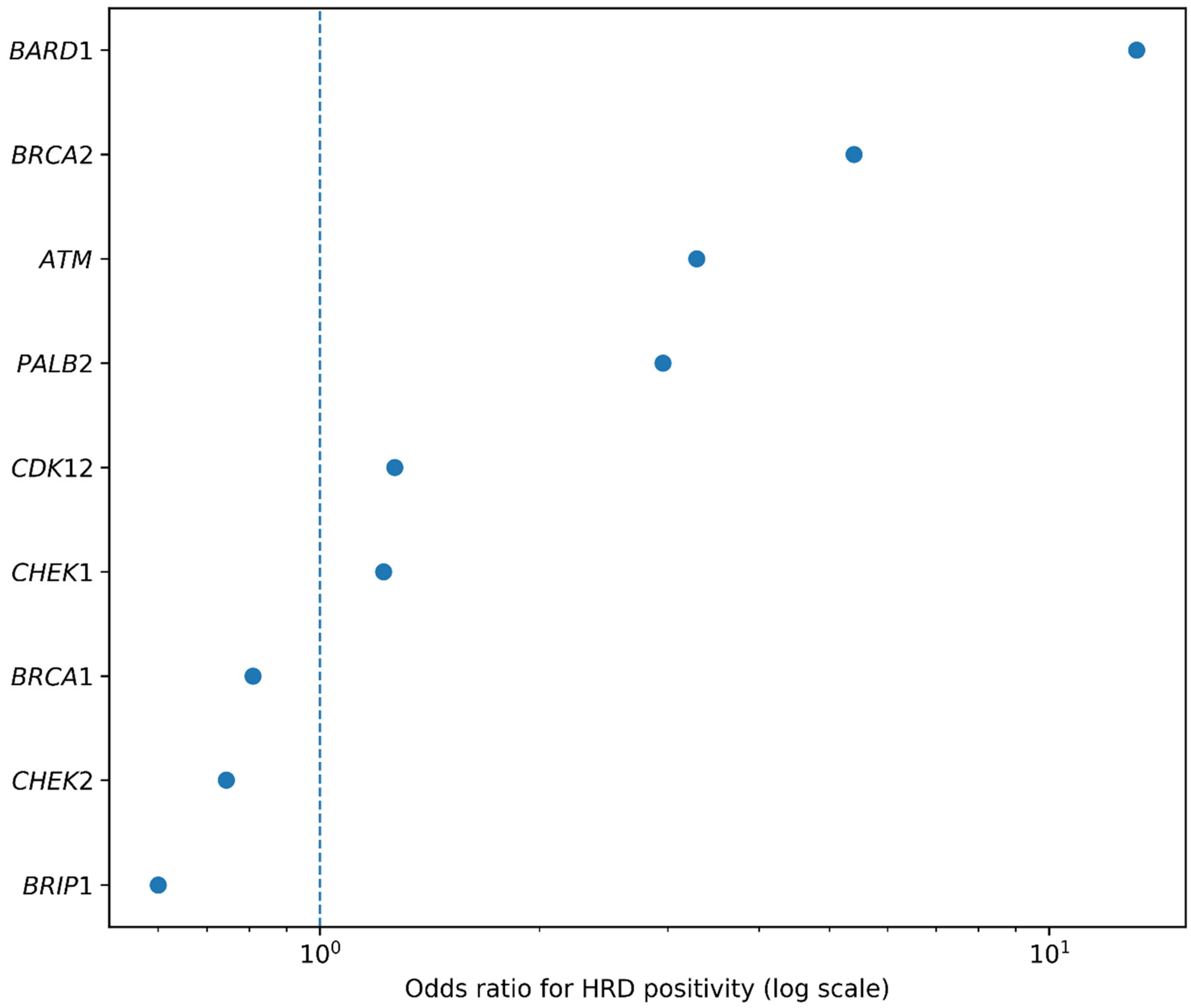

Supplementary Figure S1. Association of HRR Gene Alterations with HRD Positivity
