# Supplementary material for: LOH, LST, and TAI Profiles in Solid Tumors Harboring Pathogenic or Likely Pathogenic BRCA1/2 Variants: An Exploratory Study
Source: Int J Mol Sci. 2026 Oct 4;27(19):8851. doi: 10.3390/ijms27198851 (PMC13664783; doi:10.3390/ijms27198851)
Supplement: Supplementary file 1 [file ijms-27-08851-s001.zip › Supplementary Table S1.pdf]

**Supplementary Table S1.** Patient-level molecular and genomic scar characteristics of tumors harboring BRCA1/2 P/LP variants

| Patient ID | Tumor site | BRCA group | BRCA1/2 variant and classification | Concurrent HRR P/LP variants | LOH | LST | TAI | HRD score | LOH/ HRD | LST/ HRD | TAI/ HRD | HRD status |
|---|---|---|---|---|---|---|---|---|---|---|---|---|
| B01 | Ovary | BRCA1-only | BRCA1: c.3752_3753del, p.C1251Sfs*3 [LP] | *CDK12, CHEK2* | 0 | 13 | 18 | 31 | 0.000 | 0.419 | 0.581 | Negative |
| B02 | Ovary | BRCA1+ BRCA2 | BRCA1: c.4258C>T, p.Q1420*; BRCA1: c.3752_3753del, p.C1251Sfs*3; BRCA2: c.8021del, p.K2674Rfs*2 [P/LP/P] | *BARD1, CDK12, CHEK2, PALB2, PPP2R2A* | 19 | 29 | 18 | 66 | 0.288 | 0.439 | 0.273 | Positive |
| B03 | Breast | BRCA2-only | BRCA2: c.7976G>A, p.R2659K [P] | *ATM, BARD1, CHEK2* | 20 | 24 | 21 | 65 | 0.308 | 0.369 | 0.323 | Positive |
| B04 | Pancreas | BRCA1-only | BRCA1: c.3752_3753del, p.C1251Sfs*3 [LP] | *ATM, CHEK2* | 2 | 6 | 2 | 10 | 0.200 | 0.600 | 0.200 | Negative |
| B05 | Prostate | BRCA1-only | BRCA1: c.3752_3753del, p.C1251Sfs*3 [LP] | *CHEK2* | 5 | 5 | 3 | 13 | 0.385 | 0.385 | 0.231 | Negative |
| B06 | Rectum | BRCA1-only | BRCA1: c.3752_3753del, p.C1251Sfs*3 [LP] | *ATM, BRIP1, CDK12, CHEK1, CHEK2* | 7 | 12 | 31 | 50 | 0.140 | 0.240 | 0.620 | Negative |
| B07 | Ovary | BRCA2-only | BRCA2: c.8350C>T, p.R2784W [P] | *ATM, CHEK2* | 2 | 3 | 5 | 10 | 0.200 | 0.300 | 0.500 | Negative |
| B08 | Breast | BRCA2-only | BRCA2: c.421G>T, p.E141* [LP] | *BARD1, CHEK1, CHEK2* | 17 | 26 | 41 | 84 | 0.202 | 0.310 | 0.488 | Positive |
| B09 | Ovary | BRCA2-only | BRCA2: c.1286T>A, p.L429* [LP] | *CHEK1* | 49 | 10 | 28 | 87 | 0.563 | 0.115 | 0.322 | Positive |
| B10 | Prostate | BRCA2-only | BRCA2: c.2808_2811del, p.A938Pfs*21 [P] | *ATM, BRIP1, CDK12, CHEK2* | 2 | 13 | 8 | 23 | 0.087 | 0.565 | 0.348 | Negative |
| B11 | Ovary | BRCA1-only | BRCA1: c.3737C>A, p.T1246N [LP] | *CHEK1* | 0 | 14 | 8 | 22 | 0.000 | 0.636 | 0.364 | Negative |
| B12 | Ovary | BRCA1-only | BRCA1: c.3737C>A, p.T1246N; BRCA1: c.3333del, p.E1112Nfs*5 [LP/P] | *CHEK2* | 0 | 20 | 21 | 41 | 0.000 | 0.488 | 0.512 | Negative |
| B13 | Ovary | *BRCA1-only* | *BRCA1*: c.390C>A, p.Y130* [P] | *ATM, CHEK1* | 7 | 34 | 10 | 51 | 0.137 | 0.667 | 0.196 | Negative |

| | | | | | | | | | | | | |
|---|---|---|---|---|---|---|---|---|---|---|---|---|
| B14 | Ovary | *BRCA1-only* | *BRCA1*: c.4478_4479insA, p.E1494Gfs*7; BRCA1: c.1847_1857del, p.S616Yfs*5 [LP/LP] | *ATM, BARD1, CHEK1, RAD51B* | 17 | 21 | 29 | 67 | 0.254 | 0.313 | 0.433 | Positive |
| B15 | Small intestine | *BRCA1-only* | *BRCA1*: c.3737C>A, p.T1246N [LP] | *CHEK1, PALB2* | 10 | 17 | 26 | 53 | 0.189 | 0.321 | 0.491 | Negative |
| B16 | Ovary | *BRCA2-only* | *BRCA2:* c.1568dup, p.H523Qfs*4; BRCA2: c.4936G>T, p.E1646*; BRCA2: c.8350C>T, p.R2784W [LP/LP/P] | *CHEK1, CHEK2* | 18 | 10 | 19 | 47 | 0.383 | 0.213 | 0.404 | Negative |
| B17 | Ovary | *BRCA2-only* | *BRCA2:* c.6585_6586delinsGT, p.K2196* [LP] | *ATM, CHEK1, CHEK2, PALB2* | 20 | 11 | 15 | 46 | 0.435 | 0.239 | 0.326 | Negative |
| B18 | Ovary | *BRCA2-only* | *BRCA2*: c.7625C>T, p.T2542M [LP] | *CHEK1, CHEK2* | 0 | 8 | 10 | 18 | 0.000 | 0.444 | 0.556 | Negative |
| B19 | Breast | *BRCA2-only* | *BRCA2*: c.7007G>A, p.R2336H [P] | *ATM, CHEK1, CHEK2, PALB2* | 25 | 29 | 29 | 83 | 0.301 | 0.349 | 0.349 | Positive |
| B20 | Ovary | *BRCA2-only* | *BRCA2*: c.1796_1800del, p.S599* [P] | *CHEK1* | 26 | 33 | 30 | 89 | 0.292 | 0.371 | 0.337 | Positive |
| B21 | Colon | *BRCA1+ BRCA2* | *BRCA1:* c.4399dup, p.Q1467Pfs*9; BRCA2: c.3302A>G, p.H1101R [LP/LP] | *ATM, CHEK2* | 5 | 16 | 19 | 40 | 0.125 | 0.400 | 0.475 | Negative |
