# Supplementary material for: LOH, LST, and TAI Profiles in Solid Tumors Harboring Pathogenic or Likely Pathogenic BRCA1/2 Variants: An Exploratory Study
Source: Int J Mol Sci. 2026 Oct 4;27(19):8851. doi: 10.3390/ijms27198851 (PMC13664783; doi:10.3390/ijms27198851)
Supplement: Supplementary file 1 [file ijms-27-08851-s001.zip › Supplementary Table S2.pdf]

Supplementary Table S2. Exploratory analyses addressing BRCA1/2-HRD concordance and tumor-type heterogeneity

A) BRCA1/2 P/LP variant status and genomic HRD phenotype

BRCA1/2 P/LP variant status	HRD-positive, n (%)	HRD-negative, n (%)	Total, n (%)
Positive	7 (33.3)	14 (66.7)	21 (27.6)
Negative	9 (16.4)	46 (83.6)	55 (72.4)
Total	16 (21.1)	60 (78.9)	76 (100.0)

B) Tumor-type distribution in the BRCA1-only and BRCA2-only P/LP variant groups

Primary tumor site	BRCA1-only, n (%)	BRCA2-only, n (%)	Total, n	Fisher exact p-value
Breast	0 (0.0)	3 (30.0)	3	—
Ovary	5 (55.6)	6 (60.0)	11	—
Other tumor sites	4 (44.4)	1 (10.0)	5	—
Total	9 (100.0)	10 (100.0)	19	0.141

C) Exploratory ovarian-only analysis of relative genomic scar components

Variable	BRCA1-only (n=5), median (IQR)	BRCA2-only (n=6), median (IQR)	p-value	FDR q
LOH/HRD	0.000 (0.000-0.137)	0.338 (0.223-0.422)	0.082	0.123
LST/HRD	0.488 (0.419-0.636)	0.270 (0.219-0.353)	0.030	0.091
TAI/HRD	0.433 (0.364-0.512)	0.371 (0.329-0.476)	0.662	0.662
