# Supplementary material for: LOH, LST, and TAI Profiles in Solid Tumors Harboring Pathogenic or Likely Pathogenic BRCA1/2 Variants: An Exploratory Study
Source: Int J Mol Sci. 2026 Oct 4;27(19):8851. doi: 10.3390/ijms27198851 (PMC13664783; doi:10.3390/ijms27198851)
Supplement: Supplementary file 1 [file ijms-27-08851-s001.zip › Supplementary Table S3.pdf]

Supplementary Table S3. Association of individual HRR genes with HRD positivity

Gene	P/LP carriers	HRD+ among carriers, n (%)	OR	p-value	FDR q
BARD1	7	5 (71.4)	13.18	0.004	0.056
BRCA2	12	6 (50.0)	5.40	0.015	0.114
RAD51B	2	2 (100.0)	—	0.042	0.211
ATM	22	8 (36.4)	3.29	0.060	0.226
PALB2	13	5 (38.5)	2.95	0.132	0.395
PPP2R2A	1	1 (100.0)	—	0.211	0.526
CHEK2	47	9 (19.1)	0.74	0.773	1.000
CHEK1	35	8 (22.9)	1.22	0.782	1.000
BRCA1	11	2 (18.2)	0.81	1.000	1.000
BRIP1	7	1 (14.3)	0.60	1.000	1.000
CDK12	4	1 (25.0)	1.27	1.000	1.000
FANCL	0	0	—	1.000	1.000
RAD51C	1	0 (0.0)	0.00	1.000	1.000
RAD51D	0	0	—	1.000	1.000
RAD54L	0	0	—	1.000	1.000

P values were adjusted for multiple comparisons using the Benjamini–Hochberg false discovery rate (FDR) method. An FDR-adjusted q value <0.05 was considered statistically significant.
